# 5-[(2-Hy­droxy­eth­yl)(meth­yl)amino]­thio­phene-2-carbaldehyde

**DOI:** 10.1107/S1600536814012021

**Published:** 2014-05-31

**Authors:** Xian-Shun Sun, Nan-Qi Shao, Dan-Dan Li

**Affiliations:** a Department of Chemistry, Anhui University, Hefei 230039, People’s Republic of China; b Key Laboratory of Functional Inorganic Materials Chemistry, Hefei 230039, People’s Republic of China

## Abstract

In the title compound, C_8_H_11_NO_2_S, the aldehyde group is approximately coplanar with the thio­phene ring [maximum deviation = 0.023 (2) Å]. In the crystal, mol­ecules are linked by O—H⋯O hydrogen bonds into supra­molecular chains propagating along the *a*-axis direction.

## Related literature   

For potential applications of thio­phene derivatives, see: Encinas (2002 ▶). For a related thio­phene derivative, see: Perašínová *et al.* (2006 ▶).
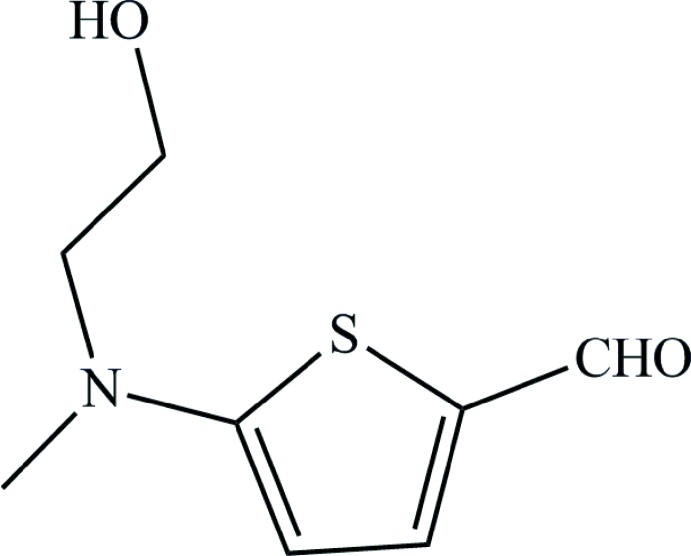


## Experimental   

### 

#### Crystal data  

C_8_H_11_NO_2_S
*M* *_r_* = 185.24
Orthorhombic, 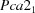

*a* = 15.764 (5) Å
*b* = 5.136 (5) Å
*c* = 11.028 (5) Å
*V* = 892.9 (10) Å^3^
*Z* = 4
Mo *K*α radiation
μ = 0.32 mm^−1^
*T* = 293 K
0.30 × 0.20 × 0.20 mm

#### Data collection  

Bruker SMART 1000 CCD area-detector diffractometer
5828 measured reflections
1564 independent reflections
1514 reflections with *I* > 2σ(*I*)
*R*_int_ = 0.020

#### Refinement  

*R*[*F* ^2^ > 2σ(*F* ^2^)] = 0.024
*wR*(*F* ^2^) = 0.067
*S* = 1.08
1564 reflections
111 parameters
1 restraint
H-atom parameters constrained
Δρ_max_ = 0.14 e Å^−3^
Δρ_min_ = −0.13 e Å^−3^
Absolute structure: Flack (1983 ▶), 756 Friedel pairs
Absolute structure parameter: −0.03 (7)

### 

Data collection: *SMART* (Bruker, 2007 ▶); cell refinement: *SAINT* (Bruker, 2007 ▶); data reduction: *SAINT*; program(s) used to solve structure: *SHELXTL* (Sheldrick, 2008 ▶); program(s) used to refine structure: *SHELXTL*; molecular graphics: *SHELXTL*; software used to prepare material for publication: *SHELXTL*.

## Supplementary Material

Crystal structure: contains datablock(s) I, Global. DOI: 10.1107/S1600536814012021/xu5786sup1.cif

Structure factors: contains datablock(s) I. DOI: 10.1107/S1600536814012021/xu5786Isup2.hkl

Supporting information file. DOI: 10.1107/S1600536814012021/xu5786Isup3.cml

CCDC reference: 996513

Additional supporting information:  crystallographic information; 3D view; checkCIF report

## Figures and Tables

**Table 1:** Hydrogen-bond geometry (Å, °)

*D*—H⋯*A*	*D*—H	H⋯*A*	*D*⋯*A*	*D*—H⋯*A*
O1—H1⋯O2^i^	0.82	1.93	2.751 (2)	174

Symmetry code: (i) 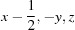.

**Fig. 1.**
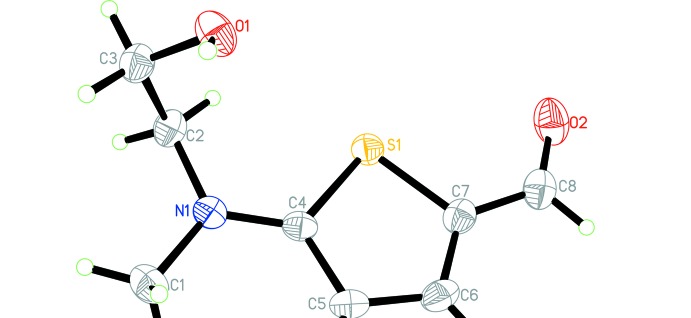
: The molecular structure of the title compound (I) showing 30% probability displacement ellipsoids.

**Fig. 2.**
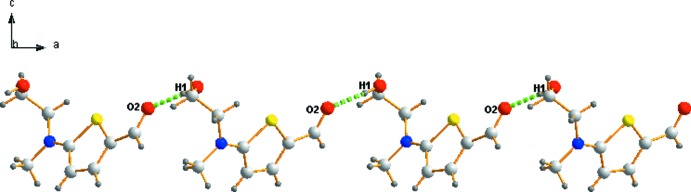
: The crystal packing of (I), showing O—H···O hydrogen-bonding interactions as dashed lines.

## supplementary crystallographic information

### 1. Comment

The introduction about the highpolarizability of sulfur atoms in thiophene rings leads to a stabilization of the conjugated chain and to excellent charge transport properties. Functional thiophene derivatives have attracted comprehensive interest among researchers all over the world and have actually been advanced to be among the most frequently used π-conjugated materials, in particular as active components in organic electronic devices and molecular electronics (Encinas *et al.*, 2002). In the title compound (I) (Fig. 1), the S1—C4 bond length of 1.7398 (18) Å is longer than the corresponding S1—C15 bond length of 1.708 (2) Å in related thiophene derivative (Perašínová *et al.* 2006), which is due to the fact that there is a higher π-electron delocalized system in the molecule 5-(fluoren-9-ylidenemethyl)thiophene-2-carbaldehyde. In the crystal structure of (I), the molecules are interconnected, *via* hydrogen bonds (Table 1) [O1—H1···O2^i^; symmetry codes: (i) *x* - 1/2, -*y*, *z*], forming a one-dimensional structure (Fig. 2).

### 2. Experimental

A 0.86 g (4.5 mmol) amount of 5-bromothiophene-2-carbaldehyde, 1.13 g (15 mmol) of 2-(methylamino)ethanol, and 0.1 g of toluene-4-sulfonic acid were mixed and stirred at a bath temperature of 373 K for 20 h. The mixture was cooled, 25 mL of water was added. The organic layer and dichloromethane extracts were combined, washed with water, and dried over MgSO_4_. Evaporation of the solvent, purification by column chromatography. ^1^H NMR: (400 Hz, DMSO-d_6_), d(p.p.m.):9.40 (s, 1H), 7.65 (d, 1H), 6.12 (d, 1H), 4.87 (t, 1H), 3.62 (q, 2H), 3.47 (t, 2H), 3.09 (s, 3H).

### 3. Refinement

All H atoms were placed in geometrically idealized positions (C—H = 0.93–0.97 Å and O—H = 0.82 Å) and allowed to ride on their parent atoms with *U*_iso_(H) = 1.2 *U*_eq_(C) or 1.5*U*_eq_(C) and *U*_iso_(H) = 1.5 *U*_eq_(O)

### Crystal data

**Table:**

C_8_H_11_NO_2_S	*F*(000) = 392
*M**_r_* = 185.24	*D*_x_ = 1.378 Mg m^−^^3^
Orthorhombic, *P**c**a*2_1_	Mo *K*α radiation, λ = 0.71069 Å
Hall symbol: P 2c -2ac	Cell parameters from 3223 reflections
*a* = 15.764 (5) Å	θ = 2.6–26.8°
*b* = 5.136 (5) Å	µ = 0.32 mm^−^^1^
*c* = 11.028 (5) Å	*T* = 293 K
*V* = 892.9 (10) Å^3^	Block, yellow
*Z* = 4	0.30 × 0.20 × 0.20 mm

### Data collection

**Table:**

Bruker SMART 1000 CCD area-detector diffractometer	1514 reflections with *I* > 2σ(*I*)
Radiation source: fine-focus sealed tube	*R*_int_ = 0.020
Graphite monochromator	θ_max_ = 25.0°, θ_min_ = 2.6°
phi and ω scans	*h* = −17→18
5828 measured reflections	*k* = −5→6
1564 independent reflections	*l* = −13→13

### Refinement

**Table:**

Refinement on *F*^2^	Secondary atom site location: difference Fourier map
Least-squares matrix: full	Hydrogen site location: inferred from neighbouring sites
*R*[*F*^2^ > 2σ(*F*^2^)] = 0.024	H-atom parameters constrained
*wR*(*F*^2^) = 0.067	*w* = 1/[σ^2^(*F*_o_^2^) + (0.0417*P*)^2^ + 0.0567*P*] where *P* = (*F*_o_^2^ + 2*F*_c_^2^)/3
*S* = 1.08	(Δ/σ)_max_ < 0.001
1564 reflections	Δρ_max_ = 0.14 e Å^−^^3^
111 parameters	Δρ_min_ = −0.13 e Å^−^^3^
1 restraint	Absolute structure: Flack (1983), 756 Friedel pairs
Primary atom site location: structure-invariant direct methods	Absolute structure parameter: −0.03 (7)

### Special details

**Table:**

Geometry. All e.s.d.'s (except the e.s.d. in the dihedral angle between two l.s. planes) are estimated using the full covariance matrix. The cell e.s.d.'s are taken into account individually in the estimation of e.s.d.'s in distances, angles and torsion angles; correlations between e.s.d.'s in cell parameters are only used when they are defined by crystal symmetry. An approximate (isotropic) treatment of cell e.s.d.'s is used for estimating e.s.d.'s involving l.s. planes.
Refinement. Refinement of *F*^2^ against ALL reflections. The weighted *R*-factor *wR* and goodness of fit *S* are based on *F*^2^, conventional *R*-factors *R* are based on *F*, with *F* set to zero for negative *F*^2^. The threshold expression of *F*^2^ > σ(*F*^2^) is used only for calculating *R*-factors(gt) *etc*. and is not relevant to the choice of reflections for refinement. *R*-factors based on *F*^2^ are statistically about twice as large as those based on *F*, and *R*- factors based on ALL data will be even larger.

### Fractional atomic coordinates and isotropic or equivalent isotropic displacement parameters (Å^2^)

**Table:**

	*x*	*y*	*z*	*U*_iso_*/*U*_eq_	
S1	0.53676 (3)	0.28672 (8)	0.22080 (5)	0.04276 (14)	
O1	0.32880 (9)	0.3310 (3)	0.35492 (15)	0.0575 (4)	
H1	0.2888	0.2479	0.3274	0.086*	
N1	0.40014 (10)	0.5376 (3)	0.13076 (14)	0.0446 (4)	
C4	0.45817 (10)	0.3498 (4)	0.11400 (16)	0.0385 (4)	
C5	0.46620 (12)	0.1849 (4)	0.01313 (18)	0.0459 (5)	
H5	0.4301	0.1889	−0.0535	0.055*	
C6	0.53411 (13)	0.0157 (4)	0.02439 (17)	0.0501 (5)	
H6	0.5479	−0.1056	−0.0351	0.060*	
C7	0.57958 (11)	0.0402 (4)	0.12977 (19)	0.0457 (4)	
O2	0.68678 (9)	−0.0828 (4)	0.26743 (18)	0.0723 (5)	
C2	0.39359 (13)	0.6822 (3)	0.24362 (18)	0.0460 (5)	
H2A	0.3870	0.8658	0.2251	0.055*	
H2B	0.4459	0.6617	0.2888	0.055*	
C1	0.33673 (14)	0.5845 (5)	0.0376 (2)	0.0583 (6)	
H1A	0.3644	0.6127	−0.0389	0.087*	
H1B	0.3040	0.7356	0.0585	0.087*	
H1C	0.2999	0.4362	0.0315	0.087*	
C8	0.64980 (13)	−0.1077 (4)	0.1692 (2)	0.0553 (5)	
H8	0.6700	−0.2351	0.1167	0.066*	
C3	0.32038 (12)	0.5959 (4)	0.32204 (19)	0.0490 (5)	
H3A	0.3184	0.7022	0.3947	0.059*	
H3B	0.2676	0.6204	0.2783	0.059*	

### Atomic displacement parameters (Å^2^)

**Table:**

	*U*^11^	*U*^22^	*U*^33^	*U*^12^	*U*^13^	*U*^23^
S1	0.0454 (2)	0.0426 (2)	0.0403 (2)	0.00002 (17)	−0.0065 (2)	−0.0035 (3)
O1	0.0509 (8)	0.0526 (8)	0.0689 (10)	−0.0042 (7)	−0.0065 (8)	0.0180 (7)
N1	0.0451 (8)	0.0484 (9)	0.0403 (7)	0.0028 (7)	−0.0043 (7)	0.0008 (7)
C4	0.0413 (10)	0.0403 (9)	0.0338 (9)	−0.0082 (7)	0.0010 (7)	0.0035 (8)
C5	0.0524 (12)	0.0537 (12)	0.0317 (9)	−0.0072 (9)	0.0002 (8)	−0.0023 (8)
C6	0.0592 (13)	0.0510 (12)	0.0400 (10)	−0.0036 (9)	0.0120 (9)	−0.0060 (9)
C7	0.0433 (10)	0.0437 (10)	0.0500 (9)	−0.0024 (8)	0.0106 (8)	0.0003 (8)
O2	0.0587 (9)	0.0613 (10)	0.0969 (13)	0.0069 (8)	−0.0212 (9)	0.0000 (9)
C2	0.0446 (9)	0.0380 (9)	0.0555 (14)	−0.0031 (7)	0.0037 (8)	−0.0050 (8)
C1	0.0518 (12)	0.0673 (15)	0.0558 (12)	0.0036 (11)	−0.0106 (9)	0.0090 (10)
C8	0.0484 (11)	0.0469 (12)	0.0705 (13)	0.0010 (10)	0.0064 (12)	0.0012 (10)
C3	0.0494 (11)	0.0427 (10)	0.0550 (12)	0.0026 (9)	0.0057 (9)	−0.0005 (9)

### Geometric parameters (Å, º)

**Table:**

S1—C4	1.7398 (18)	C7—C8	1.411 (3)
S1—C7	1.751 (2)	O2—C8	1.237 (3)
O1—C3	1.414 (3)	C2—C3	1.509 (3)
O1—H1	0.8200	C2—H2A	0.9700
N1—C4	1.342 (3)	C2—H2B	0.9700
N1—C2	1.453 (2)	C1—H1A	0.9600
N1—C1	1.454 (3)	C1—H1B	0.9600
C4—C5	1.404 (3)	C1—H1C	0.9600
C5—C6	1.384 (3)	C8—H8	0.9300
C5—H5	0.9300	C3—H3A	0.9700
C6—C7	1.371 (3)	C3—H3B	0.9700
C6—H6	0.9300		
			
C4—S1—C7	91.21 (10)	C3—C2—H2A	108.9
C3—O1—H1	109.5	N1—C2—H2B	108.9
C4—N1—C2	122.26 (16)	C3—C2—H2B	108.9
C4—N1—C1	119.38 (17)	H2A—C2—H2B	107.7
C2—N1—C1	118.15 (17)	N1—C1—H1A	109.5
N1—C4—C5	127.17 (17)	N1—C1—H1B	109.5
N1—C4—S1	121.74 (14)	H1A—C1—H1B	109.5
C5—C4—S1	111.08 (15)	N1—C1—H1C	109.5
C6—C5—C4	112.17 (18)	H1A—C1—H1C	109.5
C6—C5—H5	123.9	H1B—C1—H1C	109.5
C4—C5—H5	123.9	O2—C8—C7	125.7 (2)
C7—C6—C5	114.99 (18)	O2—C8—H8	117.1
C7—C6—H6	122.5	C7—C8—H8	117.1
C5—C6—H6	122.5	O1—C3—C2	110.98 (15)
C6—C7—C8	128.45 (19)	O1—C3—H3A	109.4
C6—C7—S1	110.53 (15)	C2—C3—H3A	109.4
C8—C7—S1	120.99 (17)	O1—C3—H3B	109.4
N1—C2—C3	113.27 (16)	C2—C3—H3B	109.4
N1—C2—H2A	108.9	H3A—C3—H3B	108.0
			
C2—N1—C4—C5	174.75 (18)	C5—C6—C7—C8	177.8 (2)
C1—N1—C4—C5	0.2 (3)	C5—C6—C7—S1	−0.2 (2)
C2—N1—C4—S1	−6.4 (2)	C4—S1—C7—C6	0.14 (15)
C1—N1—C4—S1	179.03 (15)	C4—S1—C7—C8	−178.05 (16)
C7—S1—C4—N1	−179.10 (15)	C4—N1—C2—C3	−103.3 (2)
C7—S1—C4—C5	−0.06 (15)	C1—N1—C2—C3	71.4 (2)
N1—C4—C5—C6	178.94 (18)	C6—C7—C8—O2	−177.4 (2)
S1—C4—C5—C6	0.0 (2)	S1—C7—C8—O2	0.5 (3)
C4—C5—C6—C7	0.1 (2)	N1—C2—C3—O1	60.7 (2)

### Hydrogen-bond geometry (Å, º)

**Table:**

*D*—H···*A*	*D*—H	H···*A*	*D*···*A*	*D*—H···*A*
O1—H1···O2^i^	0.82	1.93	2.751 (2)	174

Symmetry code: (i) *x*−1/2, −*y*, *z*.
